# Hsa-mir-548 family expression in human reproductive tissues

**DOI:** 10.1186/s12863-021-00997-w

**Published:** 2021-10-08

**Authors:** Ilmatar Rooda, Birgitta Kaselt, Maria Liivrand, Olli-Pekka Smolander, Andres Salumets, Agne Velthut-Meikas

**Affiliations:** 1 Department of Chemistry and Biotechnology, Tallinn University of Technology, Akadeemia tee 15, 12618 Tallinn, Estonia; 2 Competence Centre on Health Technologies, Teaduspargi 13, 50411 Tartu, Estonia; 3 Division of Obstetrics and Gynecology, Department of Clinical Science, Intervention and Technology (CLINTEC), Karolinska Institutet, 14186 Stockholm, Sweden; 4 Department of Obstetrics and Gynecology, Institute of Clinical Medicine, University of Tartu, L. Puusepa St. 8, 50406 Tartu, Estonia; 5 Institute of Genomics, University of Tartu, Riia 23b, 51010 Tartu, Estonia

**Keywords:** Hsa-mir-548 family, Hsa-miR-548ba, Granulosa cells, Myometrium, FSHR

## Abstract

**Background:**

Hsa-miR-548ba expressed in ovarian granulosa cells targets PTEN and LIFR, which are essential for ovarian follicle activation and growth. The expression pattern of hsa-miR-548ba correlates with its host gene follicle-stimulating hormone receptor (FSHR), and FSH has a positive influence on hsa-miR-548ba expression. However, hsa-miR-548ba is a member of a large hsa-mir-548 family with potentially overlapping targets. The current study aims to investigate the co-expression of hsa-mir-548 family members in FSHR-positive reproductive tissues and to explore the potential co-regulation of pathways.

**Results:**

For the above-described analysis, small RNA sequencing data from public data repositories were used. Sequencing results revealed that hsa-miR-548ba was expressed at the highest level in the ovarian granulosa cells and uterine myometrial samples together with another twelve and one hsa-miR-548 family members, respectively. Pathway enrichment analysis of microRNA targets in the ovarian samples revealed the hsa-miR-548ba and hsa-miR-548b-5p co-regulation of RAB geranylgeranylation in mural granulosa cells. Moreover, other hsa-mir-548 family members co-regulate pathways essential for ovarian functions (PIP3 activates AKT signalling and signalling by ERBB4). In addition to hsa-miR-548ba, hsa-miR-548o-3p is expressed in the myometrium, which separately targets the peroxisome proliferator-activated receptor alpha (PPARA) pathway.

**Conclusion:**

This study reveals that hsa-mir-548 family members are expressed in variable combinations in the reproductive tract, where they potentially fulfil different regulatory roles. The results provide a reference for further studies of the hsa-mir-548 family role in the reproductive tract.

**Supplementary Information:**

The online version contains supplementary material available at 10.1186/s12863-021-00997-w.

## Background

MicroRNAs (miRNAs) are a class of non-coding RNA molecules ~ 22 nucleotides in length with an important role in post-transcriptional gene expression regulation [1]. miRNAs target genes via the Watson-Crick complementarity principle. The seed sequences of miRNAs, the 2–7 nucleotides positioned in the 5′ region, play an important role in the precise targeting of mRNA [2, 3], while other regions in the miRNA sequence complement the target’s specificity [4]. Overall, miRNAs play well-established roles in gene expression regulation in normal and pathological conditions [5]. Moreover, different tissues demonstrate variable miRNA expression patterns that determine tissue characteristics, differentiation, and functions [6].

miRNAs are categorized into families according to the mature miRNA sequence and/or structure of their pre-miRNAs [7]. Mir-548 family miRNAs originate from the mariner-derived element 1 (Made1) transposable elements [8]: primate-specific short miniature inverted-repeat transposable elements (MITEs) that form almost perfect palindromes. The secondary structure of Made1 RNA contains highly stable hairpin loops that are recognized by the miRNA-processing machinery [8]. Over the course of evolution, mir-548 family members have undergone several seed-shifting events, leading to changes in the seed sequences and hence the increased variability of their mRNA targets [9].

Human miRNA hsa-miR-548ba is a member of the mir-548 family and was originally described in granulosa cells of human pre-ovulatory follicles. The hsa-miR-548ba gene is located in the intronic region of the follicle-stimulating hormone receptor (FSHR) gene [10]. Hsa-miR-548ba target analysis has revealed PTEN and LIFR as its specific targets. Both of these genes play a well-established role in follicle activation and growth, indicating that hsa-miR-548ba may also have potential regulatory importance in follicle development [11].

Follicles are ovarian structures containing the oocyte and the supporting somatic cells: theca and granulosa cells, responsible for steroidogenesis and the metabolic support of the oocyte [12]. FSHR has important functions in follicle growth in the ovaries as well as sperm development in the testes [13]. By the time the follicle reaches the pre-ovulatory stage, granulosa cells have differentiated into cumulus and mural granulosa cell populations (CGC and MGC, respectively), and the follicle is filled with follicular fluid (FF) that physically separates these cell populations [12]. The main roles of CGC and MGC are providing essential metabolic support to the oocyte and steroid hormone production, respectively [12]. FSHR knock-out mice displayed disordered follicle growth and ovulation [14]. Similarly, point mutations in human FSHR result in arrested follicle development [15]. Therefore, disturbances in FSHR expression lead to female infertility [14, 15]. Analogously, Sertoli cells in the testes express FSHR, where FSH binding indirectly activates the proliferation of germ lineage cells. FSH also regulates the role of Sertoli cells as supporters of sperm cell development [16]. Male FSHR knock-out mice and humans with point mutations in the FSHR gene have decreased spermatogenesis rates and are subfertile [17].

In addition to the ovary and testis, FSHR expression is also detected in the following reproductive tissues: the endometrium [18] and myometrium [19] of the uterus, fallopian tube [20], and cervix [19]. The uterus is mainly composed of myometrial cells, the central roles of which are protecting the growing foetus and facilitating its delivery at the end of the pregnancy through muscular contractions [21, 22]. Myometrial smooth muscle cells express receptors for estrogen and progesterone important for myometrial cell growth and tissue activation during labour [21]. In addition, FSHR is present in the myometrium, where it may also participate in myometrial contractility [23]. The endometrium is a hormonally regulated inner lining of the uterus that is receptive for embryo implantation during only a short period of the menstrual cycle. During this period, the tissue develops specific functional and structural characteristics that allow the attachment of the embryo and its implantation [24, 25].

The aim of the current study is to understand the gene regulatory network between hsa-mir-548 family members that are co-expressed in a certain cell type or tissue. Distinguishing their unique and overlapping gene targets will allow a better interpretation of their importance in tissue function. Due to the importance of FSHR in folliculogenesis and the high level of expression of hsa-miR-548ba in granulosa cells, we aimed to investigate the expression of all hsa-mir-548 members in the context of reproductive tissues where FSHR expression has been detected. We start by providing an update to the status of the hsa-mir-548 family according to the latest miRBase version [26]. Finally, we provide a model of potential co-regulation of mRNA targets and pathways between hsa-miR-548ba and other hsa-mir-548 family members in human reproductive tissues.

The mir-548 family is primate-specific. Members of this family are found in *Homo sapiens*, *Pan troglodytes*, *Callithrix jacchus*, *Macaca mulatta*, *Pongo pygmaeus* and *Gorilla gorilla*. MiR-548ba has been reported uniquely in *Homo sapiens*; therefore, this study focuses only on the members detected in humans and excludes all other primates.

## Results

**Fig. 1:**
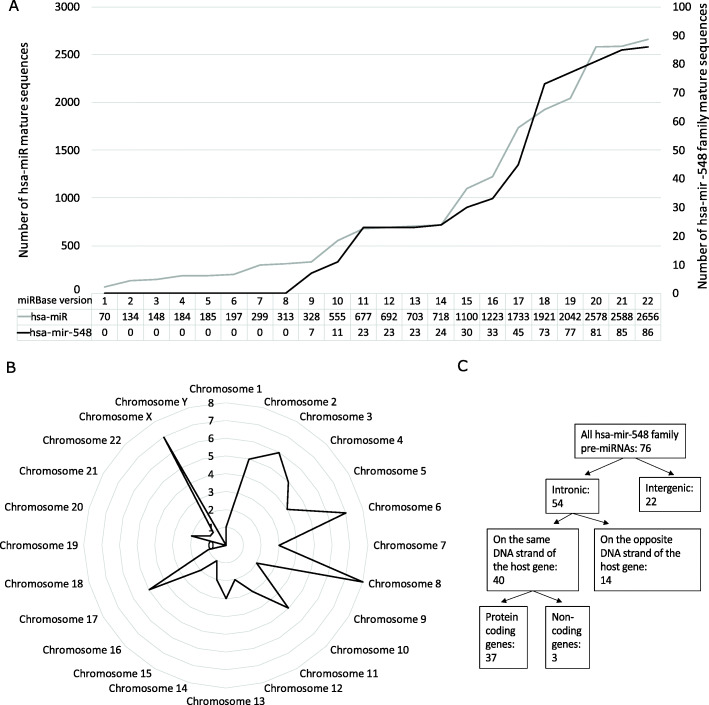
Hsa-mir-548 family members in the miRBase database and in the human genome. (**A**) The number of all annotated human miRNA (grey line) and hsa-mir-548 family (black line) sequences in the full versions of the miRBase database. (**B** and **C**) Hsa-mir-548 family pre-miRNA sequences in the human genome: distribution by chromosome (**B**), distribution between the genomic loci (**C**)

According to the full version history of miRBase, the first members of the hsa-mir-548 family were added into v9. The number of members has since been increasing with almost all new releases of miRBase in correlation with the detection of new miRNA sequences due to the increasing availability of RNA sequencing data. MiRBase v22.1 contains 86 mature human mir-548 family sequences (Fig. 1A). Hsa-miR-548ba is a relatively new member of the family, added into v20.

### Human mir-548 family distribution throughout the human genome

The human mir-548 family contains several multi-copy pre-miRNAs in the genome, and as a result, different miRNA precursor sequences give rise to the same mature sequences of hsa-miR-548. For example, hsa-miR-548f and hsa-miR-548h have 5 different pre-miRNAs in the human genome. There are in total 76 different hsa-mir-548 pre-miRNA sequences, located throughout the human genome (Fig. 1B). The highest enrichment is observed on chromosomes 6, 8, and X. Two human chromosomes (19 and Y) lack hsa-mir-548 family sequences completely.

From the 76 hsa-mir-548 sequences, 54 are located in the intronic regions and 22 sequences are of intergenic origin (Fig. 1C). From 54 intronic miRNAs, 40 sequences were located on the same DNA strand as their host gene (Additional file 1 Supplementary Table S1). As 37 of these 40 are protein-coding genes, there is a potential co-transcription of the host gene and the corresponding intronic miRNA.

### Sequence similarity analysis of hsa-mir-548 family members

**Fig. 2:**
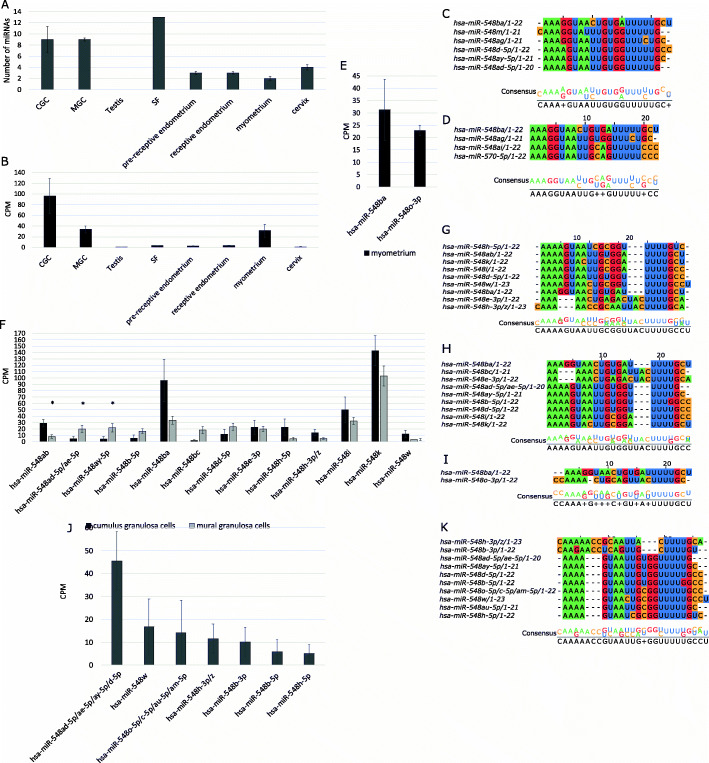
The expression and sequence similarity of hsa-mir-548 family members in reproductive tissue samples. (**A**) The average number of hsa-mir-548 family members present in reproductive tissues with cut-off > 10 counts per million (CPM). (**B**) The expression levels of hsa-miR-548ba in reproductive tissues. The sequencing results are displayed as a mean of CPM ± SEM. CGC–cumulus granulosa cells (*n* = 3), MGC–mural granulosa cells (n = 3), testis (*n* = 5), SF–seminal fluid (*n* = 1), pre-receptive endometrium (*n* = 12), receptive endometrium (n = 12), myometrium (n = 3), and cervix (*n* = 4). (**C**) The closest hsa-mir-548 family members to hsa-miR-548ba according to sequence similarity. (**D**) Hsa-mir-548 family members which share a seed sequence with hsa-miR-548ba. The sequence length in nucleotides is noted after the slash. (**E**) miRNAs from the hsa-miR-548 family expressed in the myometrium. (**F)** miRNAs of the hsa-miR-548 family expressed in cumulus and mural granulosa cells. The alignment of hsa-miR-548 family sequences co-expressed with hsa-miR-548ba in the analysed tissues: (**G**) cumulus granulosa cells; (**H**) mural granulosa cells; (**I**) myometrium; (**J**) The expression of hsa-mir-548 family members in the cell-depleted follicular fluid of the ovarian follicle; (**K**) the alignment of extracellular miRNAs observed in follicular fluid. Expression levels are displayed as a mean of counts per million (CPM) ± SEM). *p < 0.05, Student’s t-test

Sequence similarity analyses were performed for both mature and pre-miRNA sequences. Shorter distances between mature sequences on the phylogenetic tree indicate higher conservation compared to pre-miRNA sequences (Additional file 2 Supplementary Fig. S1 and S2). The hsa-miR-548ba mature sequence displayed the shortest distances to the following miRNAs: hsa-miR-548 m, hsa-miR-548ag, hsa-miR-548d-5p, hsa-miR-548ay-5p, and hsa-miR-548ad-5p (Fig. 2C, Additional file 2 Supplementary Fig. S1). In addition, hsa-miR-548ag; hsa-miR-548ai and hsa-miR-570-5p share the critical seed sequence with hsa-miR-548ba (Fig. 2D), although the two latter miRNAs demonstrate dissimilarities in their 3’part and therefore reside more distantly in the phylogenetic tree (Additional file 2 Supplementary Fig. S1).

Moreover, a sequence similarity analysis was performed between hsa-mir-548 family members and Made1, the MITE elements giving rise to these miRNAs (Additional file 2 Supplementary Fig. S3 and S4, respectively). Hsa-miR-548-5p sequences demonstrate higher conservation and similarity to Made1 compared to hsa-miR-548-3p sequences. This result confirms a previous similar observation [9]. Hsa-miR-548ba belongs to the hsa-miR-548-5p sequences and is therefore a more conserved family member. Hence, it is highly probable that hsa-miR-548ba and other hsa-mir-548 family members co-expressed in a tissue regulate a set of the same mRNA targets.

### Hsa-mir-548 family expression in reproductive tissues

In order to quantify the expression levels of the hsa-miR-548 family members in human reproductive tissues, small RNA high-throughput sequencing results from male and female reproductive tissues were analysed (Table 1). Tissues were selected for analysis according to the availability of small RNA sequencing data and positivity for FSHR expression. From the male reproductive tissues, small RNA sequencing results were only available for the whole testis tissue homogenate and seminal fluid (SF). From the female reproductive tissues, data was available for MGC, CGC, and FF of the ovary, myometrium, and endometrium from the uterus and cervix.

**Table 1:** A description of data used in the hsa-mir-548 family analysis of reproductive tissues

**Cellular samples**	**Tissue of origin**	**Cell type**	**Data repository**	**Accession number**	**Number of samples**	**Description**
	Ovary	Granulosa	GEO	GSE46508	6	Two cell types of human granulosa cell samples were obtained from pre-ovulatory follicles: cumulus granulosa (CGC) and mural granulosa (MGC) collected from stimulated pre-ovulatory follicles [10].
	Uterus	Endometrial tissue	GEO	GSE108966	24	Human endometrial samples were collected from two time-points of the same menstrual cycle: early-secretory phase corresponding to pre-receptive endometrium and mid-secretory phase corresponding to receptive endometrium [27].
	Uterus	Myometrium tissue	GEO	GSE100338	3	[28]
	Uterus	Cervix tissue	GEO	GSE145372	4	[29]
	Testis	Testis tissue	GEO, ENCODE	ENCSR229WIW, ENCSR626GVP and GSE149084	5	Whole testis tissue sections [30].
	Reproductive track	Non-sperm cellular fraction of seminal fluid (SF)	GEO	GSE56686	1	Non-sperm cellular fraction of SF, which includes prostatic epithelial, urothelial and inflammatory cells [31].
**Extracellular samples**	**Tissue of origin**	**Cell type**	**Data repository**	**Accession number**	**Number of samples**	**Description**
	Ovary	Follicular fluid (FF)	GEO	GSE157037	8	Extracellular miRNAs were extracted from cell-depleted ovarian follicular fluid (FF) from stimulated pre-ovulatory follicles [32].

The highest number of hsa-mir-548 family members was detected from the SF, CGC, and MGC samples (cut-off > 10 counts per million (CPM), Fig. 2A). From the testis samples, none of the hsa-mir-548 family members reached the set cut-off limit (Fig. 2A). The full lists of hsa-mir-548 family members expressed above > 10 CPM cut-off level are presented in Additional file 1 Supplementary Table S2.

The highest expression levels of hsa-miR-548ba were observed in ovarian CGC and MGC samples (Fig. 2B). Outside of the ovary, hsa-miR-548ba expression was the highest in myometrial tissue compared to the other samples. Testicular, SF, endometrial, and cervical samples demonstrated expression levels with borderline detection values (Fig. 2B).

### Hsa-mir-548 expression in granulosa cells and myometrium

To further study the potential and significance of the post-transcriptional co-regulation effect that hsa-miR-548ba may exhibit with its co-expressed family members, ovarian and myometrial samples were further analysed, as hsa-miR-548ba was only detected in these samples.

Sequencing results of granulosa cells revealed the expression of 13 different mature hsa-mir-548 family members (Fig. 2F). From those miRNAs, three (hsa-miR-548ab, hsa-miR-548ad-5p/ae-5p and hsa-miR-548ay) were differentially expressed between MGC and CGC samples (*p* < 0.05).

The miRNAs which share the same seed sequence with hsa-miR-548ba (Fig. 2D) are not co-expressed in granulosa cells. However, sequence alignment results reveal that a number of miRNAs detected in granulosa cells share the seed sequence with each other (Fig. 2G-H). Specifically, hsa-miR-548ab, hsa-miR-548d-5p, hsa-miR-548 h-5p, hsa-miR-548i, and hsa-miR-548w in CGC and hsa-miR-548ay, hsa-miR-548ae-5p, hsa-miR-548ad-5p, hsa-miR-548b-5p, hsa-miR-548d-5p and hsa-miR-548i in MGC contain the same seed sequences. Therefore, the co-regulation of common target genes by these miRNAs is possible and expected to occur in granulosa cells.

From miRNAs with the highest sequence similarity to hsa-miR-548ba, only hsa-miR-548ay-5p and hsa-miR-548ad-5p are present in MGC, and hsa-miR-548d-5p in both CGC and MGC are expressed above the cut-off > 10 CPM (Fig. 2F-H).

Compared to granulosa cells, the myometrium expresses only two hsa-mir-548 family members above the > 10 CPM cut-off: hsa-miR-548ba and hsa-miR-548o-3p (Fig. 2E). Hsa-miR-548o-3p is evolutionarily distant from hsa-miR-548ba, and these two miRNAs do not share a common seed sequence (Fig. 2I). In addition to the myometrium, hsa-miR-548o-3p is expressed in the endometrium, cervix, and SF samples (Additional file 1 Supplementary Table S2).

### Extracellular hsa-mir-548 family miRNAs in the follicular fluid

miRNAs are known to be present in the extracellular space as a part of RNA-binding protein (RBP) complexes or as loaded into extracellular vesicles (EV) [33]. However, the potential for the hsa-mir-548 family miRNAs to be secreted into extracellular spaces has not been studied. Due to the high expression levels of hsa-miR-548ba and several other members of the family in the ovarian follicular somatic cells, the extracellular profile was determined from the example of the ovarian cell-depleted FF [22], where seven family members were detected (50% of samples > 10 CPM cut-off, Fig. 2J). We observed that the miRNAs expressed at the highest levels in the cellular samples (hsa-miR-548k, hsa-miR-548ba and hsa-miR-548i) were not detected in FF. This suggests that those miRNAs are cell-specific and are not secreted into extracellular spaces. On the other hand, hsa-miR-548o-5p, hsa-miR-548c-5p, hsa-miR-548am-5p, and hsa-miR-548b-3p in FF are not expressed in granulosa cells above the determined cut-off level (Fig. 2J).

Specific motifs in the 3′ half of the miRNA sequence have the potential to determine whether miRNAs are secreted into the extracellular space or are retained in the cells: for example, GGAG and UGCA appear frequently in extracellular and cellular miRNAs, respectively [34]. In addition, the AGG motif may be involved in extracellular miRNA trafficking [35]. However, miRNAs present in FF samples do not contain GGAG nor AGG motifs (Fig. 2K). Cellular motif UGCA is present in hsa-miR-548 h-3p/z. The fact that those miRNAs are present in the extracellular space may be the result of non-specific secretion.

### The signature of hsa-mir-548 family expression is characteristic for each female reproductive tissue

**Fig. 3:**
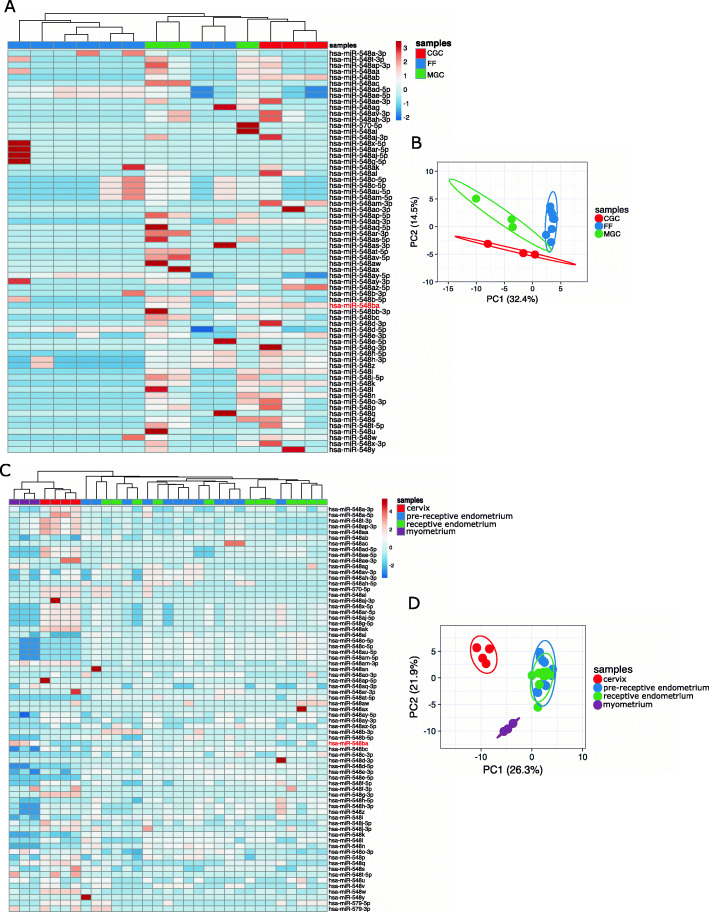
Expression levels of hsa-mir-548 family miRNAs in the human ovarian follicle and uterine samples. (**A**) A heatmap of hsa-mir-548 family expression levels in individual ovarian samples; (**B**) a PCA plot of ovarian follicle cellular and extracellular samples according to the expression levels of hsa-mir-548 family miRNAs; (**C**) a heatmap of hsa-mir-548 family expression levels in individual uterine samples; (**D**) a PCA plot of uterine samples according to the expression levels of hsa-mir-548 family levels. FF–cell-depleted follicular fluid, CGC–cumulus granulosa cells, MGC–mural granulosa cells. The location of hsa-miR-548ba on the heatmap is highlighted in red. The heatmap colour scale displays ln(x + 1) transformed CPM values

All ovarian follicle sample types form separate clusters according to their hsa-mir-548 family expression patterns (Fig. 3A-B). As expected, cellular and extracellular samples cluster separately. Moreover, two granulosa cell types form separate clusters according to their hsa-mir-548 expression patterns (Fig. 3B). MGC and FF samples display more similar expression patterns compared to CGC cells (Fig. 3A-B). This may indicate that MGC is the primary source of hsa-mir-548 members secreted into FF as MGC is the most abundant somatic cell type inside the pre-ovulatory follicle.

In addition to granulosa cells, hsa-miR-548ba exhibited high expression levels in the myometrial tissue. For clustering analysis, all available uterine tissue samples (endometrium, myometrium and cervix) were compared. The results exhibited a characteristic hsa-mir-548 family expression pattern for endometrium, myometrium, and cervical samples (Fig. 3C-D). Endometrial samples of pre-receptive and receptive stages clustered together (Fig. 3C-D), indicating that hsa-mir-548 family expression levels do not significantly change upon acquiring endometrial receptivity. Moreover, myometrial and cervical samples form closer clusters compared to endometrial samples (Fig. 3C).

Overall, the clustering analysis illustrates that it is possible to distinguish female reproductive tissues and cell types by the expression signature of the hsa-mir-548 family members. Therefore, this miRNA family possesses regulatory roles specific to cell type.

### Pathways regulated by hsa-miR-548 members co-expressed in granulosa cells

**Fig. 4:**
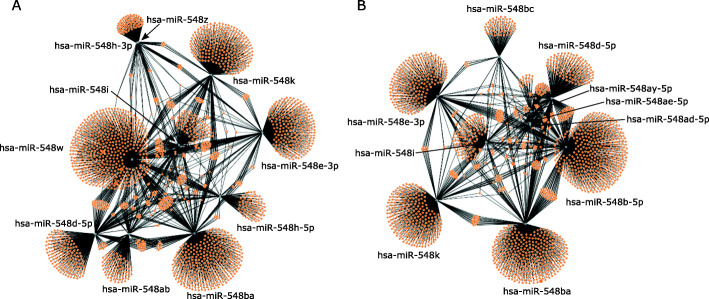
Target prediction for hsa-mir-548 family miRNAs expressed in cumulus granulosa cells (**A**) and mural granulosa cells (**B**). Each orange node represents one target gene, and genes targeted by more than one miRNA are connected with an edge

Since multiple hsa-mir-548 family members are co-expressed with hsa-miR-548ba in the ovarian granulosa cells, we investigated their tissue-specific potential for regulating common signalling pathways with relevance to female fertility. Target genes were predicted for all miRNAs expressed above > 10 CPM cut-off level in CGC or MGC cells and the obtained lists were used as inputs for Reactome pathway enrichment analysis, the results of which are presented in Additional file 1 Supplementary Table S3. Target prediction results revealed that, despite the similarities between the sequences, miRNAs expressed in CGC or MGC target mostly individual genes with a small overlapping part (Fig. 4A and B, respectively).

Pathway enrichment analysis of targeted genes concluded that hsa-miR-548ba does not co-regulate common pathways in CGC with other cell-type-specific hsa-miR-548 family members. In MGC, hsa-miR-548ba revealed the co-regulation of “RAB geranylgeranylation” pathway with hsa-miR-548b-5p. From the other hsa-mir-548 family members hsa-miR-548d-5p and hsa-miR-548i co-regulate, “PIP3 activates the AKT signalling” pathway in both CGC and MGC. This pathway is additionally targeted by hsa-miR-548w and hsa-miR-548b-5p in CGC and MGC, respectively. Additionally, “PI5P, PP2A, and IER3 regulate PI3K/AKT signalling” pathway is commonly regulated by hsa-miR-548d-5p and hsa-miR-548b-5p in MGC (Additional file 1 Supplementary Table S3A and S3B).

In the context of ovarian function, the above-mentioned pathways “PIP3 activates AKT signalling” and “PI5P, PP2A and IER3 regulate PI3K/AKT signalling” have been previously studied [36–38]. In addition, the “Translocation of SLC2A4 (GLUT4) to the plasma membrane” targeted by hsa-mir-548ba in both CGC and MGC, as well as “Signalling by ERBB4” targeted by hsa-miR-548b-5p in MGC, demonstrate the importance of the corresponding miRNAs in ovarian functions [39, 40].

### Pathways regulated by hsa-miR-548 members expressed in myometrium

Although the myometrial cells exhibited the expression of only two hsa-mir-548 family members (hsa-miR-548ba and hsa-miR-548o-3p), a commonly regulated pathway “Signalling by BRAF and RAF fusions” by these two miRNAs was detected.

In the context of myometrial functions, the following important regulatory pathways were targeted by hsa-miR-548o-3p: “PI5P, PP2A and IER3 regulate PI3K/AKT signalling” and “PPARA activates gene expression”.

## Discussion

Hsa-mir-548 is a primate-specific miRNA family derived from Made1 transposable element [8]. Made1 elements are MITEs with genomic locations either close to or within genes, where they may be involved in gene regulation [41]. Hsa-mir-548 family members are transcribed from most human chromosomes, while some other miRNA families exhibit chromosome-specific locations in the genome [7]. The distribution analysis of hsa-mir-548 family members in the human genome exhibited that the majority of pre-miRNA sequences (54/76) are located in the intronic regions of genes. This is in an accordance with the preferable genome locations of MITEs [41]. Moreover, chromosome Y, which contains the smallest number of genes compared to other chromosomes [42], does not contain any hsa-mir-548 members. However, gene-rich chromosome 19 [43] likewise did not contain any hsa-mir-548 miRNA sequences. It is possible that not all hsa-mir-548 family members have been discovered. Since the first sequence of hsa-mir-548 was included to miRBase, new sequences have been added to almost every new miRBase release, correlating with the rapid development and reduced cost of high-throughput sequencing technologies. Moreover, using a bioinformatic approach, 34 additional precursor sequences of hsa-mir-548 have been discovered, indicating that this family could be larger [44]. However, their expression still needs experimental validation.

miRNAs are important gene expression regulators in both the male and female reproductive tissues and aberrant miRNA expression can lead to infertility [45, 46]. Hsa-miR-548ba expression analysis in reproductive samples revealed that this particular miRNA is expressed at the highest level in granulosa cells, where it was first discovered. It has been previously shown that the expression pattern of hsa-miR-548ba is similar to that of FSHR and FSH treatment upregulates hsa-miR-548ba expression levels in human granulosa cells [11]. Human ovarian granulosa cells [13], endometrium [47], myometrium [19], cervix [19] and testis Sertoli cells [13] all express FSHR. In addition to granulosa cells, hsa-miR-548ba exhibited high expression levels in the myometrial tissue. These results reveal the tissue-specific expression of hsa-miR-548ba that may be derived from a different miRNA expression regulation than that observed in the granulosa cells of its host gene, FSHR. However, differences in expression level may also be caused by technical errors. The datasets used for this study were obtained from data repositories and, therefore, RNA extraction and library preparation were not universal for all samples. This may be the cause of the lower expression of hsa-miR-548ba in the endometrium, cervix, and testis samples.

Overall, granulosa cells express 13 members of the hsa-mir-548 family. From those, miRNAs hsa-miR-548ab, hsa-miR-548ad-5p/ae-5p, and hsa-miR-548ay-5p were differentially expressed between MGC and CGC samples (*p*-value < 0.05). Although detected in a few other human sample types (hsa-miR-548ab expression is reported in human B-cells [48], hsa-miR-548ad-5p/ae-5p and hsa-miR-548ay-5p are present in synovial tissue samples [49], and hsa-miR-548ad-5p is present in blood plasma samples), the roles of these specific miRNAs have not been investigated. However, in our samples, hsa-miR-548ad-5p was detected in extracellular FF as one of the most abundant miRNAs. According to the comparisons in ovarian datasets, it can be deduced that hsa-miR-548ad-5p is secreted from MGC and may be involved in intercellular signalling in the follicle. Therefore, the investigated miRNA family potentially has unknown importance in follicular function.

miRNAs which share the same seed sequence with hsa-miR-548ba are not co-expressed in granulosa cells, which indicates that this miRNA potentially has an individual specific regulatory role in the ovary. However, despite this, some targets were shared between other co-expressed members of the hsa-mir-548 family with different seed sequences. It has been well established that one mRNA can be targeted by multiple miRNAs [50]. Moreover, target prediction algorithms like miRWalk use additional features to miRNA seed sequence for target prediction [51]. The hsa-mir-548 family has gone through several seed-shifting events, which has resulted in various seed sequences in the members [9]. Different seed sequence variants were also present in miRNAs expressed in ovarian and myometrial samples. Nevertheless, some hsa-mir-548 family miRNAs expressed in granulosa cells have common seed sequences and consequently overlap with part of the predicted target genes; for example, hsa-miR-548b-5p, hsa-miR-548d-5p, and hsa-miR-548i expressed in MGCs.

The majority of enriched pathways were targeted by different individual miRNAs in the granulosa cells. All together there were three exceptions. The first exception was the “PIP3 activates AKT signaling” pathway, which is targeted by hsa-miR-548d-5p and hsa-miR-548i in CGC and MGC and additionally targeted by hsa-miR-548w and hsa-miR-548b-5p in CGC and MGC, respectively. This pathway is involved in regulating the balance between dormancy and activation of follicles, granulosa cell differentiation, and proliferation [38]. The second exception was the “PI5P, PP2A, and IER3 regulate PI3K/AKT signalling” pathway targeted by hsa-miR-548d-5p in CGC and MGC, and hsa-miR-548b-5p in MGC. IER3 is a part of a gonadotropin-EGR2-IER3 axis with a role in granulosa cell survival during follicle development [36]. Additionally, PP2A participates in the regulation of PKC-mediated inflammation in rat granulosa cells [37]. The last co-regulated pathway is “RAB geranylgeranylation” targeted by hsa-miR-548ba and hsa-miR-548b-5p in MGC. The depletion of the geranylgeranylation substrate geranylgeranyl diphosphate (GGPP) in mice oocytes inhibits Rab27a geranylgeranylation, which is required for Rab protein activation. Rab27a plays a possible role in oocyte protein secretion. Therefore, disturbances in this pathway impair oocyte-granulosa cell communication, which is necessary for normal follicle development [52].

Pathways targeted by individual hsa-mir-548 members have additional known roles in granulosa cells. For example, hsa-miR-548ba targets the “translocation of SLC2A4 (GLUT4) to the plasma membrane” pathway. GLUT4 is involved in glycose uptake and FSH stimulates this process in granulosa cells [39]. Granulosa cells of polycystic ovarian syndrome patients have a tendency to display abnormal glycose metabolism. Therefore, normal glycose metabolism is important for granulosa cell function [39]. Hsa-miR-548b-5p targets “Signalling by ERBB4” in MGC. ERBB4 plays a role in normal follicle development and disturbances in ERBB4 levels may lead to ovarian dysfunction [40]. To conclude, in addition to hsa-miR-548ba, other hsa-mir-548 family members regulate pathways important for granulosa cell functions.

Pathways “PIP3 activates AKT signalling” and “PI5P, PP2A and IER3 Regulate PI3K/AKT signalling” are targeted by miRNAs which share the seed sequences (hsa-miR-548d-5p, hsa-miR-548b-5p, hsa-miR-548i, and hsa-miR-548w). However, additional family members expressed in granulosa cells have the same seed sequence but do not target those pathways. Alignment results of miRNAs present in granulosa cells and alignment of the whole miRNA family demonstrated that, in addition to seed shifting events, nucleotide substitutions are present in miRNA sequences. These molecular events have changed potential targeting features [4] and have led to different target genes between miRNAs with the same seed sequence.

Myometrial samples express two hsa-mir-548 members: hsa-miR-548ba and hsa-miR-548o-3p. Both miRNAs regulate one common pathway: “Signalling by BRAF and RAF fusions”. BRAF and RAF fusion is a result of chromosomal rearrangement events and is detected in distinct cancer types [53]. Therefore, in normal myometrial tissue, this pathway is not present. In addition, hsa-miR-548o-3p targets the “PI5P, PP2A, and IER3 regulate PI3K/AKT signalling” and “PPARA activates gene expression” pathways. From the first targeted pathway, PP2A regulates proteins involved in smooth muscle contraction [54]. In the second pathway, PPARA levels increase in the late pregnancy myometrium (gestation range 20–35 weeks) compared to nonpregnant women and decrease by the time of labour, suggesting that PPARA plays a role in maintaining pregnancy [55]. This indicates that the hsa-mir-548 family may have a regulatory role in myometrial gene expression regulation involved in contractile functions. Moreover, hsa-miR-548o-3p expression was not detected in ovarian samples but was present in endometrial and cervical samples, confirming its organ-specific expression.

miRNAs have been detected from all body fluids, including FF [32, 56], and may be involved in cell-to-cell communication [33]. miRNAs can be secreted from cells as a part of RBPs or packed into EVs [33]. Many possible sorting mechanisms are proposed for loading miRNAs into EVs: sequence characteristics, post-transcriptional modifications, subcellular location, and intracellular concentration [33]. In this study, FF which contains both RBPs and EVs, was searched for hsa-mir-548 family members. As a result, 7 miRNAs were detected in FF. Some of the miRNAs were only detected in FF and not in granulosa cells, for example, hsa-miR-548o-5p and hsa-miR-548c-5p. miRNAs expressed at the highest levels in cellular samples were not present in FF, indicating that the secretion mechanism is not based on the intercellular concentration of miRNA molecules. miRNAs present in extracellular samples were aligned and a possible export motif was searched for. Known miRNA secretion motifs GGAG [34] and AGG [35] are not present in the miRNA sequences of hsa-mir-548 family members present in FF. Nevertheless, hsa-mir-548 family members have been detected from other body fluids in addition to FF: hsa-miR-548b-5p, hsa-miR-548c-5p and hsa-miR-548i [57], and hsa-miR-548a-3p [58] in blood serum samples, hsa-miR-548b-3p in blood plasma, bronchial lavage and peritoneal fluid, and hsa-miR-548d-5p in amniotic fluid [59]. Some miRNAs infiltrate into the FF from blood plasma [32], explaining the lack of their expression in the granulosa cells. Additionally, the oocyte has not been investigated as the source of miRNAs secreted into the FF due to the lack of such human data. Therefore, hsa-mir-548 family members are secreted into extracellular space by other cell types as well as ovarian granulosa cells. The mechanism by which hsa-mir-548 family members are selected for secretion remains unknown.

## Conclusion

From all the analysed FSHR-positive samples, hsa-miR-548ba transcribed from the intronic region of FSHR gene can be detected in the ovarian granulosa cells and the myometrium. This suggests that the expression of hsa-miR-548ba and FSHR are differently co-regulated in other FSHR-positive tissues. In addition to hsa-miR-548ba, twelve and one other hsa-mir-548 family members are expressed in granulosa and myometrium samples, respectively. Moreover, hsa-mir-548 family members are detectable from the extracellular ovarian FF. miRNA target pathway enrichment analysis revealed that hsa-miR-548ba and hsa-miR-548b-5p co-regulate the RAB geranylgeranylation pathway in MGC. Disturbances in this pathway impair oocyte-granulosa cell communication. In addition to hsa-miR-548ba, other family members separately regulate essential pathways for granulosa cell function (PIP3 activates AKT signalling and signalling by ERBB4). This reveals that hsa-mir-548’s family regulatory role in granulosa cells is wider than previously acknowledged. Moreover, hsa-miR-548o-3p expressed in myometrium targets the PPARA pathway which is associated with the maintenance of pregnancy. Furthermore, hsa-miR-548o-3p presents uterine-specific expression as it was detected only in myometrial, endometrial and cervical samples. Overall, hsa-mir-548 family members may play regulatory roles in ovarian follicle activation, development, granulosa cell differentiation, and proliferation. In the myometrium, the hsa-mir-548 family was predicted to regulate myometrial contractility and has a potential importance in the maintenance of pregnancy.

## Methods

### Hsa-mir-548 family members and sequences

The analysis of hsa-mir-548 family member curation was performed using the miRBase database [26]. Information about miRNA mature sequences was downloaded from all full miRBase versions with the exception of v22.1, which is the current release. Genomic locations of pre-miRNA sequences in the human genome were obtained from NCBI Gene [60] and Ensembl [61] databases.

Mature and pre-miRNA sequences were aligned in Jalview (v2.11.0) [62] using the Clustal Omega algorithm with standard settings [63]. Phylogenetic trees of mature and pre-miRNA sequences were constructed with the neighbour-joining method [64] from reads aligned with Clustal Omega in Jalview.

### Hsa-mir-548 family expression in human reproductive tissues

All sequencing data used in the analyses were previously published and available in open data repositories (Table 1). From all available data, only samples from healthy control subjects were used. All miRNA raw FASTQ files were quality-filtered with Trimmomatic v0.39 [65] with the options of SLIDINGWINDOW:2:20. Adapter sequences were removed and reads below 17 nucleotides in length were discarded. The remaining filtered and trimmed reads were counted and mapped to the primary assembly of human genome GRCh38 and annotated miRNA sequences from miRBase v22.1 using miRDeep2 with standard settings [66]. All miRNA raw counts obtained from miRDeep2 results were normalized to counts per million (CPM) using the edgeR package v.3.28.1 [67]. miRNA results were filtered by expression levels, and the cut-off was set > 10 CPM for all cellular samples. The cut-off for extracellular samples was set to > 10 CPM in 50% of samples. Data visualization on heatmap and PCA plots was performed in ClustVis [68]. Statistical significance between CGC and MGC was calculated via a two-tailed Student’s t-test. The statistical significance level was set at *p* < 0.05.

### Target prediction and gene ontology analysis

Target genes were predicted for miRNAs with an expression cut-off level of > 10 CPM with miRWalk version 3 [51]. Obtained miRNA target lists were input for gene enrichment analysis with miRWalk pathway analysis tool, and a statistical significance threshold was set at Benjamini-Hochberg FDR < 0.1.

## Supplementary Information

**Additional file 1: Supplementary Table S1.** Human hsa-mir-548 family pre-miRNA sequence locations in human genome. **Supplementary Table S2.** Hsa-mir-548 family miRNAs expressed in reproductive tissues. **Supplementary Table S3.** Reactome pathways of predicted miRNA targets

**Additional file 2: Supplementary Fig. S1.** Phylogenetic tree of mature sequences of hsa-mir-548 family members. **Supplementary Fig. S2.** Phylogenetic tree of pre-miRNA sequences of miR-548 family members. **Supplementary Fig. S3.** Phylogenetic tree of Made1 and hsa-mir-548 family members. **Supplementary Fig. S4.** The alignment of Made1 and hsa-mir-548 family mature sequences

## Data Availability

The datasets analysed during the current study are available in the Gene Expression Omnibus under accession numbers: GSE46508 (https://www.ncbi.nlm.nih.gov/geo/query/acc.cgi?acc=GSE46508), GSE108966 (https://www.ncbi.nlm.nih.gov/geo/query/acc.cgi?acc=GSE108966), GSE100338 (https://www.ncbi.nlm.nih.gov/geo/query/acc.cgi?acc=GSE100338), GSE145372 (https://www.ncbi.nlm.nih.gov/geo/query/acc.cgi?acc=GSE145372), GSE149084 (https://www.ncbi.nlm.nih.gov/geo/query/acc.cgi?acc=GSE149084), GSE56686 (https://www.ncbi.nlm.nih.gov/geo/query/acc.cgi?acc=GSE56686) and GSE157037 (https://www.ncbi.nlm.nih.gov/geo/query/acc.cgi?acc=GSE157037) and ENCODE repository under accession numbers: ENCSR229WIW (https://www.encodeproject.org/experiments/ENCSR229WIW/) and ENCSR626GVP (https://www.encodeproject.org/experiments/ENCSR626GVP/).

## Abbreviations

CGC: cumulus granulosa cells
CPM: counts per million
EV: extracellular vesicles
FF: follicular fluid
FSHR: follicle-stimulating hormone receptor
Made: mariner-derived element 1
MGC: mural granulosa cells
MITEs: miniature inverted-repeat transposable elements
RBP: RNA-binding protein
SF: seminal fluid
