# Supplementary material for: Hsa-mir-548 family expression in human reproductive tissues
Source: BMC Genom Data. 2021 Oct 8;22:40. doi: 10.1186/s12863-021-00997-w (PMC8501715; doi:10.1186/s12863-021-00997-w)
Supplement: Supplementary file 2 — Additional file 2: Supplementary Fig. S1. Phylogenetic tree of mature sequences of hsa-mir-548 family members. Supplementary Fig. S2. Phylogenetic tree of pre-miRNA sequences of miR-548 family members. Supplementary Fig. S3. Phylogenetic tree of Made1 and hsa-mir-548 family members. Supplementary Fig. S4. The alignment of Made1 and hsa-mir-548 family mature sequences [file 12863_2021_997_MOESM2_ESM.docx]

**Supplementary Figures**

**Phylogenetic tree of mature sequences of hsa-mir-548 family members**


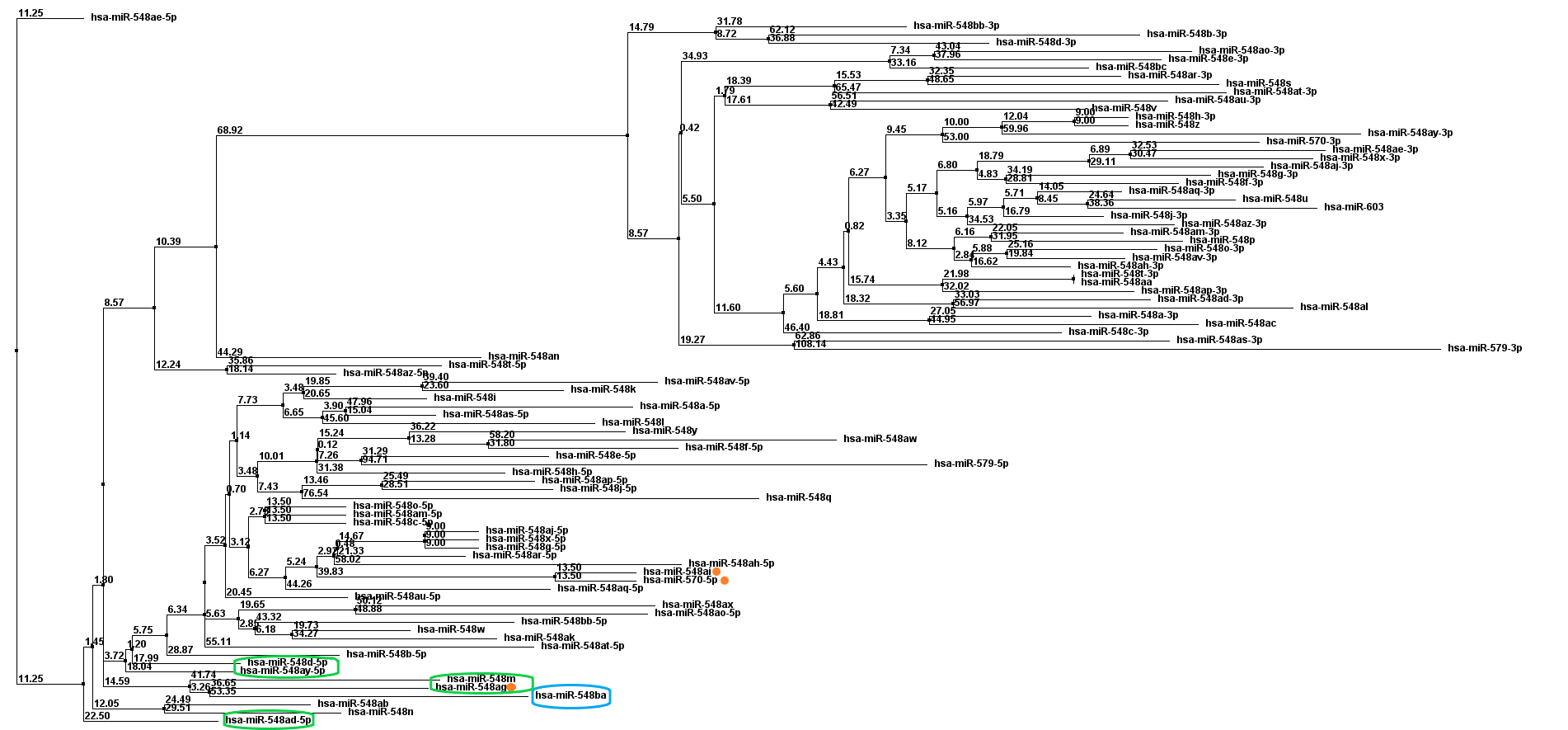


**Supplementary Figure 1.** Phylogenetic tree of mature sequences of hsa-mir-548 family members. The location of hsa-miR-548ba is marked in blue, closest members to the hsa-miR-548ba are marked in green and members which share same seed sequence with hsa-miR-548ba are marked with an orange dot.

**Phylogenetic tree of pre-miRNA sequences of miR-548 family members**


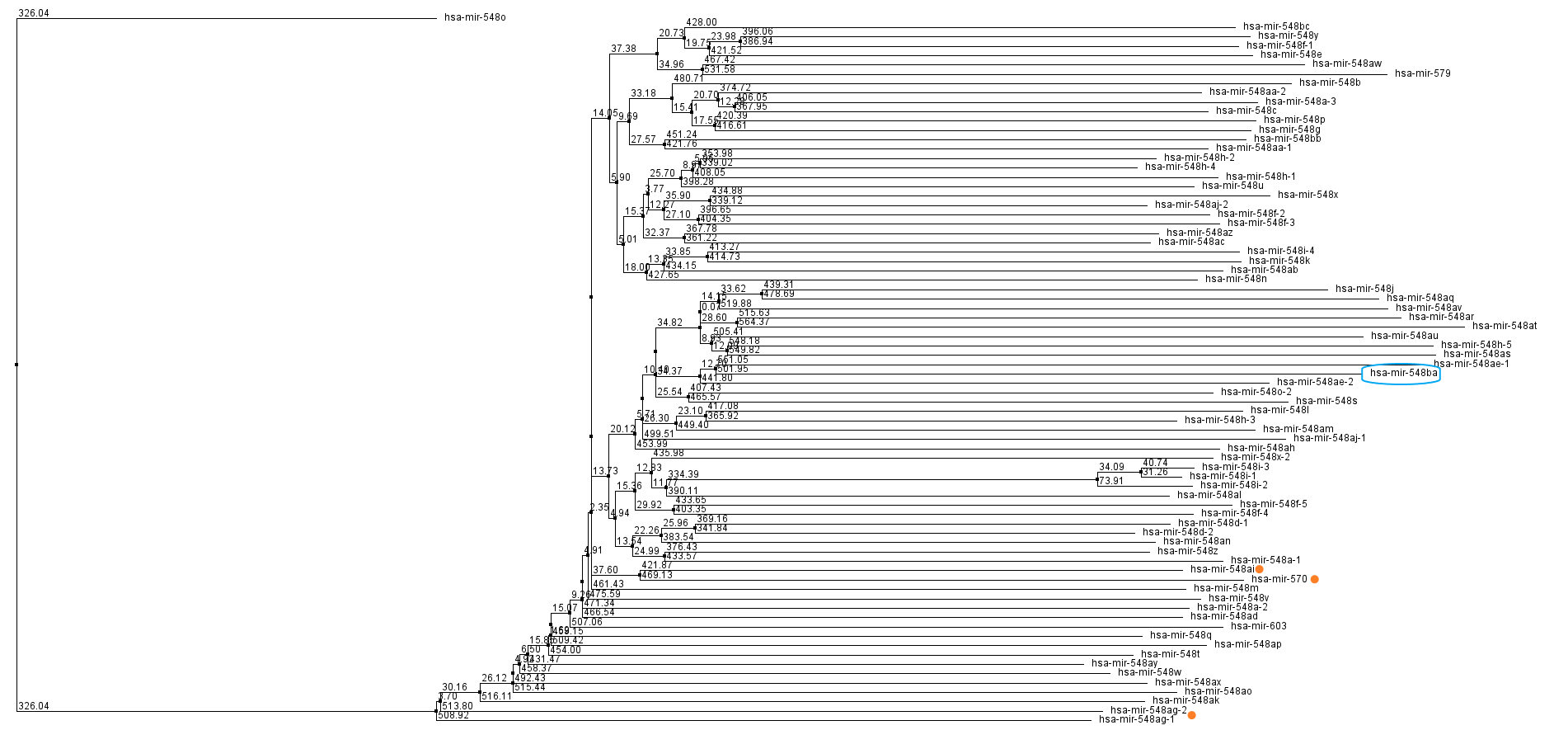


**Supplementary Figure 2.** Phylogenetic tree of pre-miRNA sequences of miR-548 family members. The location of hsa-miR-548ba is marked in blue and members which share the same seed sequence with hsa-miR-548ba are marked with orange dot.

**Phylogenetic tree of Made1 and hsa-mir-548 family members**

**
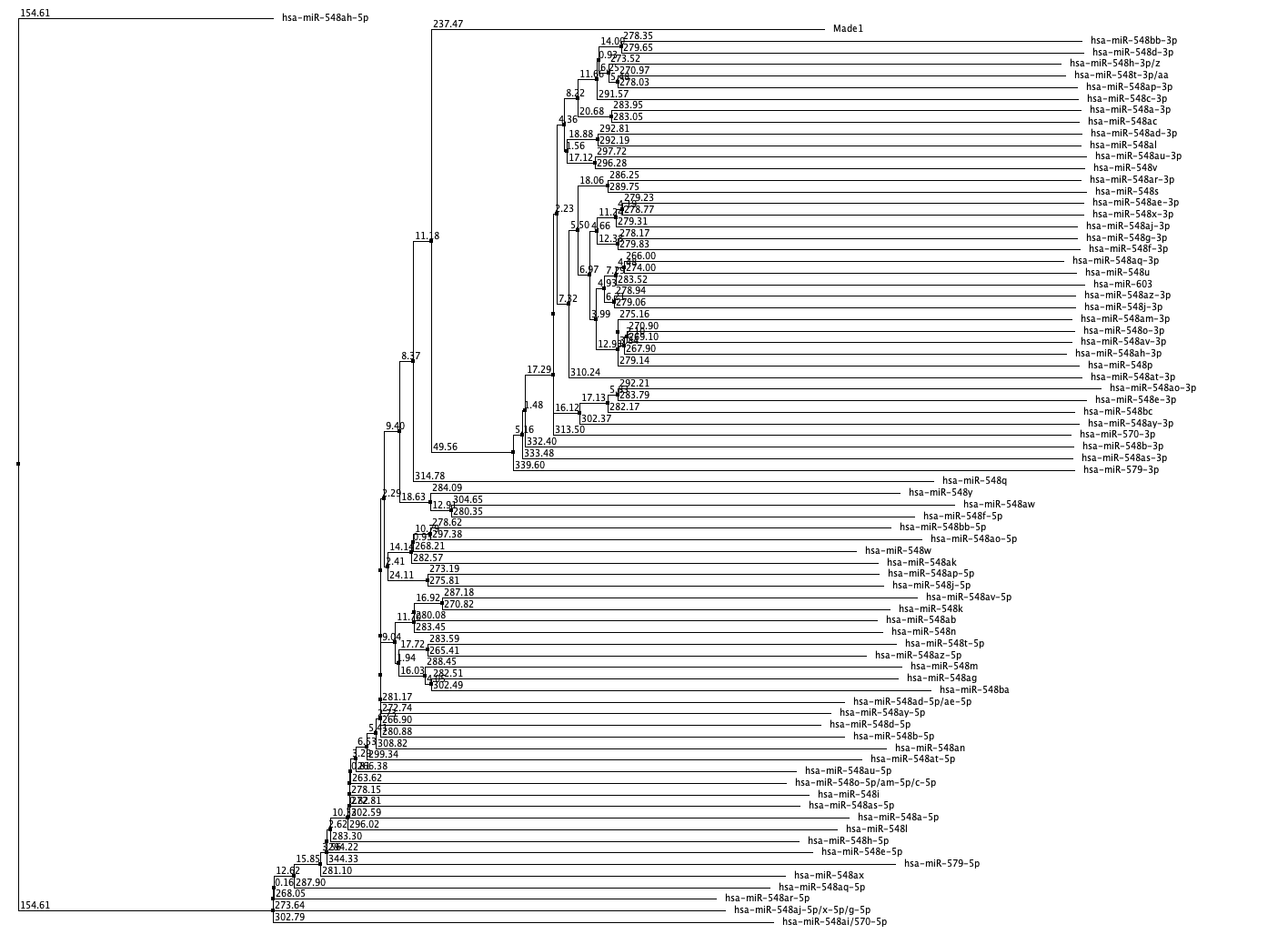
**

**Supplementary Figure 3.** Phylogenetic tree of Made1 and mature sequences of hsa-mir-548 family members.

**The alignment of Made1 and hsa-mir-548 family mature sequences**

**
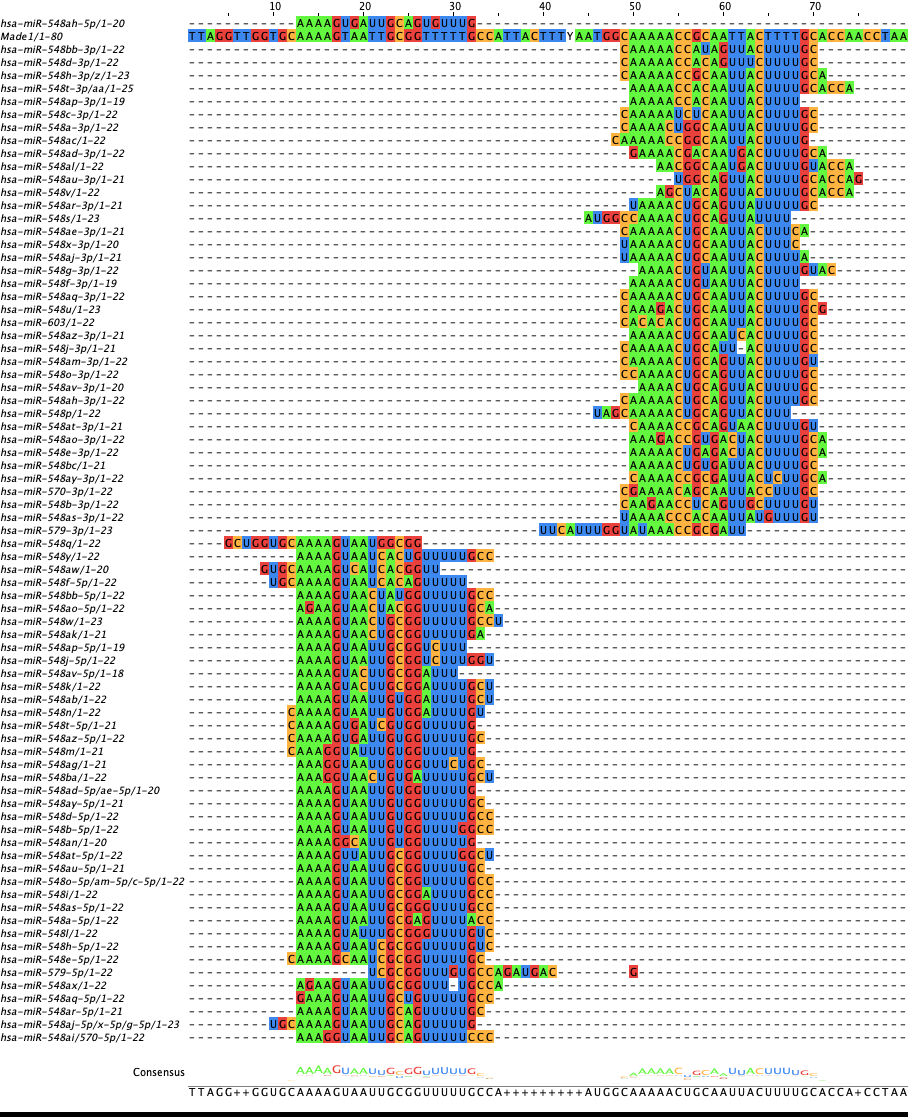
**

**Supplementary Figure 4.** The alignment of Made1 and hsa-mir-548 family mature sequences. Sequences are sorted by phylogenetic tree and miRNAs with the same mature sequences are collapsed together.
